# Increased Expression of hsa_circ_0002111 and Its Clinical Significance in Papillary Thyroid Cancer

**DOI:** 10.3389/fonc.2021.644011

**Published:** 2021-02-26

**Authors:** Gongbo Du, Runsheng Ma, Hongqiang Li, Jiao He, Kaixiang Feng, Dongpeng Niu, Detao Yin

**Affiliations:** ^1^Department of Thyroid Surgery, The First Affiliated Hospital of Zhengzhou University, Zhengzhou, China; ^2^Key Discipline Laboratory of Clinical Medicine Henan, Zhengzhou, China; ^3^Academy of Medical Sciences, Zhengzhou University, Zhengzhou, China

**Keywords:** circular RNA, hsa_circ_0002111, papillary thyroid carcinoma, biomarker, therapeutic target

## Abstract

Circular RNA (circRNA) is a newly discovered non-coding RNA. Recent reports suggest that circRNAs are key regulators of tumorigenesis because of their special structure. In order to investigate the role of hsa_circ_0002111 in papillary thyroid cancer (PTC), we use quantitative real-time polymerase chain reaction (qRT-PCR) to determine the expression pattern of hsa_circ_0002111 in 82 paired PTC and adjacent non-cancerous thyroid tissues. Cell counting kit-8, colony formation, and transwell assays were conducted to assess the effect of hsa_circ_0002111 on PTC cell proliferation, migration, and invasion. We found that the expression of hsa_circ_0002111 was significantly up-regulated in PTC tissues compared with adjacent non-cancerous tissues (P < 0.0001). Expression of hsa_circ_0002111 was also associated with advanced TNM stage and lymph-node metastasis of patients with PTC. The area under the receiver operating characteristic curve was 0.833. Further, cell function assays showed that hsa_circ_0002111 inhibition significantly suppressed the proliferation and invasion abilities of PTC cells *in vitro*. In conclusions, the study findings show that the over-expression of hsa_circ_0002111 promotes PTC, and thus hsa_circ_0002111 may be a potential diagnostic biomarker and therapeutic target for PTC.

## Introduction

Thyroid carcinoma (TC) is the most common endocrine cancer, and its morbidity rate has increased over the past several decades (1, 2). Papillary thyroid carcinoma (PTC) represents the major histopathologic type of TC, and accounts for approximately 90% of all TC cases (3). Although the overall 5-year survival rate of PTC is 97%, the outcomes after curative surgical resection are not favorable for patients in advanced stages (4). Poor clinical prognosis of advanced PTC cases is partly due to an incomplete understanding of the molecular mechanisms underlying PTC occurrence and development. Thus, it is crucial to identify new biomarkers and therapeutic targets to improve the diagnosis and treatment of PTC.

Circular RNAs (circRNAs), which were discovered approximately four decades ago (5), have attracted considerable attention in recent years (6, 7). circRNAs, which occur in low abundance, are non-coding RNAs (ncRNAs) that were initially considered as products of mis-splicing, or as by-products of pre-mRNA processing (8). In contrast with other types of ncRNAs, circRNAs form covalently closed, continuous loops with neither a 5′-to-3′ polarity nor a poly-adenylated tail. Further, they are not sensitive to digestion by RNases; consequently, they are more conserved and stable by nature (9). Owing to the closed structure of circRNAs, they are likely to be promising and effective biomarkers for cancers, and can possibly function as molecular targets for cancer treatment.

In this study, we found a circRNA hsa_circ_0002111(circBase ID: hsa_circ_0002111) is located on chromosome 8p22 and is a transcript product of *PSD3* gene by bioinformatics analysis. And based on data from the GEO datasets, hsa_circ_0002111 was one of the most up-regulated circRNAs in PTC tissues and was markedly up-regulated in PTC tissue samples compared with its expression in matched normal tissues. Thus, we chose hsa_circ_0002111 to explore the role of it. We investigated the association between hsa_circ_0002111 expression and clinical features of patients with PTC. Additionally, *in vitro* function assays were used to explore the roles of hsa_circ_0002111 in PTC progression.

## Materials and Methods

### Patient Tissues

From March 2020 to July 2020, 82 PTC tissue and adjacent normal tissue samples were collected from 82 patients who underwent radical surgery at the First Affiliated Hospital of Zhengzhou University, Zhangzhou, China. This study was reviewed by the Ethics Review Committees of the First Affiliated Hospital of Zhengzhou University (2020-KY-392) and all patients provided written informed consent. All tissue samples were immediately snap-frozen and stored in liquid nitrogen until further use. The patients had not received chemotherapy, radiotherapy, or immunotherapy before their surgical procedure.

### Quantitative Real-Time Polymerase Chain Reaction (qRT-PCR)

Total RNA was extracted from paired PTC tissues and adjacent non-tumor tissues using the TRIzol™ Reagent (Invitrogen, California, USA) by following the manufacturer’s instructions. RNA was reverse-transcribed into cDNA using the RevertAid First Strand cDNA Synthesis Kit (Takara, Kyoto, Japan) according to the manufacturer’s instructions. Each reaction for quantitative analysis was performed in a total volume of 25 μL, including 2× qPCR Mix (12.5 μL), gene primers (7.5 μM/2.0 μL each), cDNA (2.5 μL), and ddH_2_O (8 μL) as per the instructions of the SYBR^®^ Green Fast qPCR Mix (Servicebio, Wuhan, China). β-actin was used as the reference standard for each cDNA sample. The thermal cycling conditions were as follows: 95°C for 10 min, followed by 40 cycles at 95°C for 15 s and 60°C for 60 s. The melting curve was obtained from 60 to 95°C with a temperature increase of 0.3°C per 15 s. The primer sequences were as follows: β-actin forward, 5′- GTGCCAAAATGCTCAAGGAAAT-3′, β-actin reverse, 5′-GAAGGGCAGCTTTCTTTGTGAC-3′, hsa_circ_0002111 forward, 5′-AGGGGTAAATGAGGGTGTTGAT-3′, and hsa_circ_0002111 reverse, 5′- TGGCTGCTTCCACATTGCTG-3′. The relative expression of hsa_circ_0002111 was calculated using the 2^−ΔΔCt^ method.

### Bioinformatics Analysis

The microarray data of circRNA profiles in PTC tissues and paired non-tumor tissues were retrieved in NCBI GEO Datasets (http://www.ncbi.nlm.nih.gov/gds, No. GSE93522). Normalized microarray data were analyzed using GEO2R after applying log2 transformation. GEO2R, an interactive web tool, allows for comparison of original submitter-supplied processed data (https://www.ncbi.nlm.nih.gov/geo/geo2r/).

### Cell Culture and Transfection

Nthy-ori 3-1 cell line is an immortalized human thyroid follicular epithelial cell line derived from normal adult thyroid tissue that has been transfected with a plasmid containing origin-defective SV40 genome (SV-ori). TPC-1 and B-CPAP cell lines are differentiated PTC cell lines derived from the tumor tissue of adult woman with PTC. All of these cell lines were purchased from Shanghai Cell Biochemical Institute (Shanghai, China). The cells were cultured in the Roswell Park Memorial Institute (RPMI)-1640 medium with 10% foetal bovine serum (FBS; Gemini, California, USA) in a humidified incubator with 5% CO_2_ at 37°C.

Based on the circBase information of hsa_circ_0002111, the lentivirus which packaged the small interfering RNA(siRNA) targeting the hsa_circ_0002111(si-circ_0002111) and negative control-siRNA (si-NC) was constructed by Genechem (Shanghai, China). For cell transfection, TPC-1 and B-CPAP cells were cultured at 2×10^5^ cells/well in 6-well plates. After the cells attached to the wall, 10 µl lentivirus with a titer of 2×10^8^ TU/ml (MOI=10) and 40 µl HitransG P infection enhancer (Genechem, Shanghai, China) were added into each well. At 12 h, the culture medium was replaced with RPMI-1640 medium containing 10% FBS. The cells were screened by culture medium containing 2.5 µg/ml puromycin (PerkinElmer, Massachusetts, USA). After 48 h, culture medium containing 1.5 µg/ml puromycin was used to screen for ≥ 2 weeks to obtain a stable transfected cell line.

### Cell Counting Kit-8 (CCK-8) Assays

Suspensions of digested cells in the logarithmic growth phase were uniformly seeded in 96-well plates at a density of 2×10^3^ cells/well, and established in three replicate wells. Then, 4, 24, 48, and 72 h later, 10 µL of the CCK-8 detection reagent (Dojindo, Kumamoto, Japan) was added to each well. The culture plate was incubated at 37°C for 2 h, and then placed in a Universal Microplate Reader to measure the absorbance at 450 nm.

### Colony Formation Assay

Cells were seeded in a culture dish at a density of 500 cells/well, and were incubated for 2 weeks. Thereafter, the cells were washed with phosphate-buffered saline (PBS), fixed with paraformaldehyde, and stained with crystal violet. The number of colonies was counted and calculated using the ImageJ software.

### Transwell Invasion Assay

The transwell invasion assay was performed with 24-well culture plates and by matching transwell upper chamber inserts (Corning, New York, USA). Briefly, 2 × 10^4^ cells were mixed with 200 μL serum-free RPMI-1640 medium, and were seeded in the upper chamber inserts pre-coated with Matrigel matrix (BD, New Jersey, USA). Then, 600 μL medium containing 10% FBS was added to the lower chamber. After incubating at 37°C for 24 h, the cells on the upper membrane surface were wiped off using a cotton swab, and the cells that had traversed the membrane were stained with crystal violet. Finally, the cells were counted using an inverted microscope (Olympus, Tokyo, Japan), and the images were evaluated using the ImageJ software.

### Wound Healing Assay

Cells transfected with siRNAs were cultured in a 6-well plate at a density of 5 × 10^5^ cells/well. Sterile tips were used to scratch each well when the cells were tightly attached to the well surface. Next, the suspended cell fragments were removed by washing with sterile PBS, and the cells were cultured in a serum-free RPMI-1640 medium for 24 h. Then, images of the migrating cells were captured using an inverted microscope (Olympus, Tokyo, Japan).

### Statistical Analysis

All statistical analyses were performed using the SPSS 25.0 software (IBM, USA). Comparisons between two groups were performed using the Student’s *t*-test. The relationship between hsa_circ_0002111 and clinical features of PTC was analyzed using the chi-squared (χ^2^) test. Receiver operating characteristic curves (ROC) were generated to evaluate the diagnostic value of hsa_circ_0002111 for PTC. P < 0.05 indicated a statistically significant difference.

## Results

### hsa_circ_0002111 Expression Is Up-Regulated in PTC Tissues and Cell Lines

**Figure 1:**
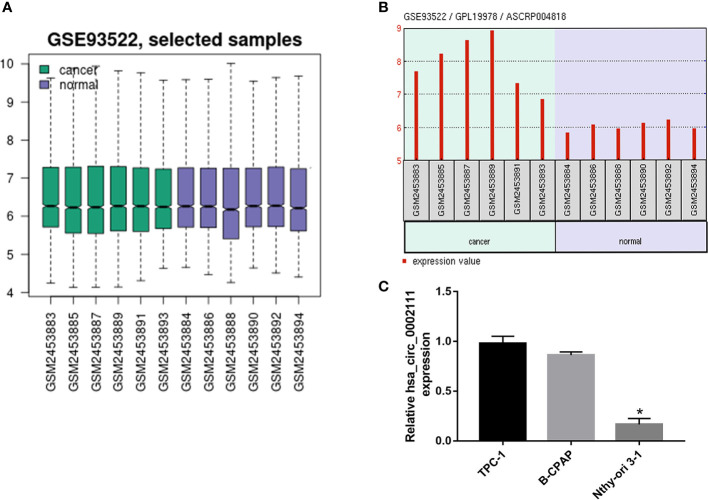
Circular RNA (circRNA) expression profile in papillary thyroid cancer (PTC). **(A)** Box plot showed the normalized intensities from the tumor and non-tumor tissue samples **(B)** Gene Expression Omnibus dataset, GSE93522, showed that hsa_circ_0002111 was significantly increased in tumor tissues compared to that in non-tumor tissues. **(C)** Quantitative real-time polymerase chain reaction (qRT-PCR) verification of the expression of hsa_circ_0002111 in TPC-1, B-CPAP, and Nthy-ori 3-1 cell lines. *P < 0.05.

**Figure 2:**
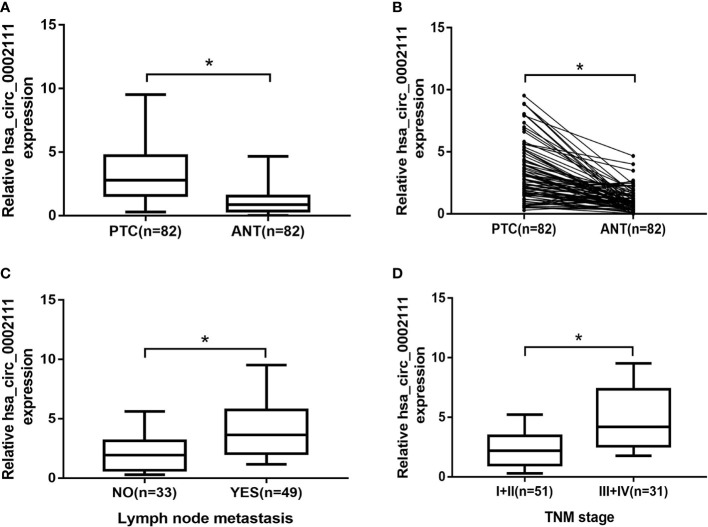
Expression level of hsa_circ_0002111 in papillary thyroid cancer (PTC) tissues and its clinical significance. **(A, B)** hsa_circ_0002111 expression was significantly higher in PTC tissues compared to that in adjacent non-tumor tissues. **(C)** hsa_circ_0002111 expression was higher in PTC patients with lymph-node metastasis. **(D)** hsa_circ_0002111 expression was higher in PTC patients with III + IV stage compared to that in patients with I + II stage. *P < 0.05.

To investigate the relationship between circRNAs and PTC, we analyzed the GSE93522 dataset by using GEO2R after applying log2 transformation. Box plots showed normalized intensities from the tumor and non-tumor tissue samples (**Figure 1A**). Microarray data showed that hsa_circ_0002111 was the second up-regulated circRNAs in PTC tissues and the expression of hsa_circ_0002111 was markedly up-regulated in six PTC tissue samples compared with its expression in matched normal tissues (**Figure 1B**). Thus, we selected hsa_circ_0002111 for further investigations. qRT-PCR results showed that hsa_circ_0002111 expression was higher in the PTC cell lines (B-CPAP and TCP-1) than that in the Nthy-ori 3-1 cell line (**Figure 1C**; P < 0.05). We further explored the expression of hsa_circ_0002111 in 82 samples of PTC tissues and adjacent non-tumor tissues by qRT-PCR, and observed that hsa_circ_0002111 expression was significantly up-regulated in PTC tissues than that in the matched normal tissues (**Figures 2A, B**; P < 0.05).

### hsa_circ_0002111 Up-Regulation Is Associated With Clinical Features of PTC

We also analyzed hsa_circ_0002111 expression in different TNM stages of PTC samples, and in lymph-node metastatic or lymph-node non-metastatic PTC samples, and observed that increased hsa_circ_0002111 expression was significantly correlated with an advanced TNM stage (III-IV) and lymph-node metastasis (**Figures 2C, D**; P < 0.05). **Table 1** shows that hsa_circ_0002111 expression was significantly associated with TNM stage (P =0.04) and lymph-node metastasis (P = 0.013). Particularly, there was a positive correlation between the expression of hsa_circ_0002111 and lymph-node metastasis. However, there were no significant correlations between hsa_circ_0002111 expression and other clinical features, such as gender, age, and tumor size (P > 0.05).

**Table 1:** Correlation between hsa_circ_0002111 expression and clinicopathological features of papillary thyroid cancer (PTC).

Variables	No. of cases	hsa_circ_0002111 expression		p-value
		High	Low	
Gender				0.618
Male	22	10	12	
Female	60	31	29	
Age (years)				0.499
<55	49	26	23	
≥55	33	15	18	
Tumor size (cm)				0.073
<2	48	20	28	
≥2	34	21	13	
Lymph node metastasis				0.013
No	33	11	22	
Yes	49	30	19	
TNM stage				0.040
I–II	51	21	30	
III–IV	31	20	11	

### Potential Diagnostic Value of hsa_circ_0002111 in PTC

**Figure 3:**
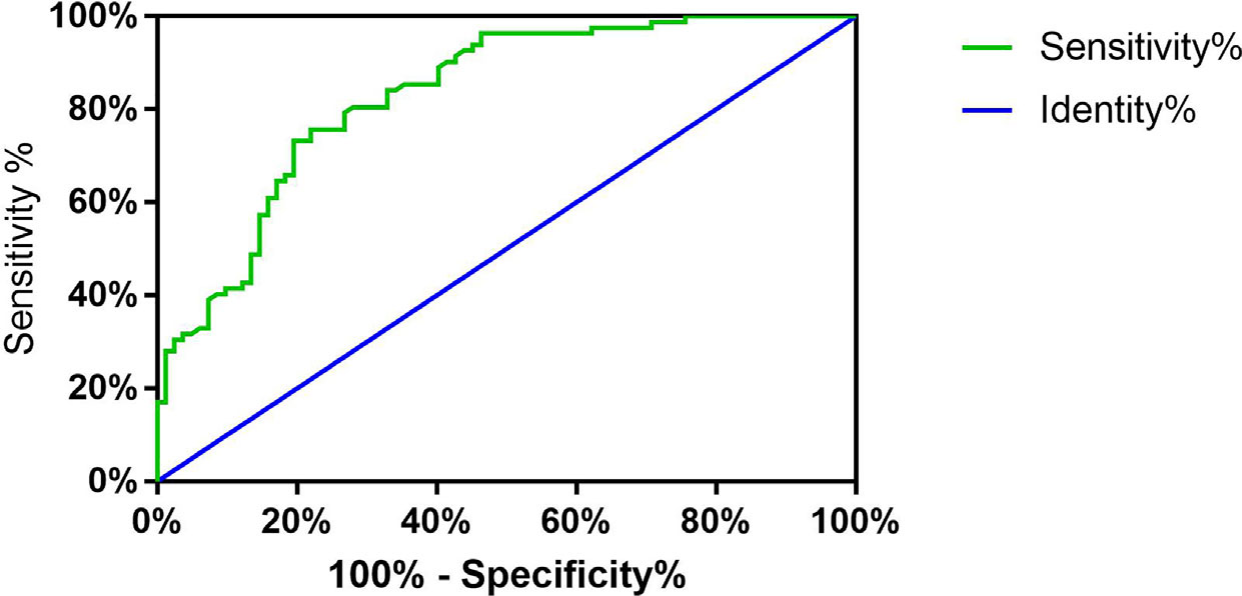
Receiver operator characteristic (ROC) curve. Analysis of sensitivity and specificity of hsa_circ_0002111 as a novel tumor marker for papillary thyroid cancer (PTC) by ROC curve and the area under the ROC curve was 0.833 (95% CI = 0.7713–0.8935; P < 0.0001).

Next, we explored the diagnostic value of hsa_circ_0002111 as a potential biomarker for PTC by using an ROC curve. The ROC curve was generated to evaluate whether hsa_circ_0002111 could be utilized as a novel tumor marker for PTC. As shown in **Figure 3**, the area under the ROC curve (AUC) was 0.833 (95% CI = 0.7713 to 0.8935; P < 0.0001), suggesting that the expression level of hsa_circ_0002111 could serve as a potential, diagnostic tumor marker for PTC. Moreover, the Youden index, sensitivity, and specificity were 0.561, 75.6, and 80.5%, respectively, using the cut-off value of 1.525.

### hsa_circ_0002111 Inhibition Suppresses PTC Cell Proliferation and Invasion

**Figure 4:**
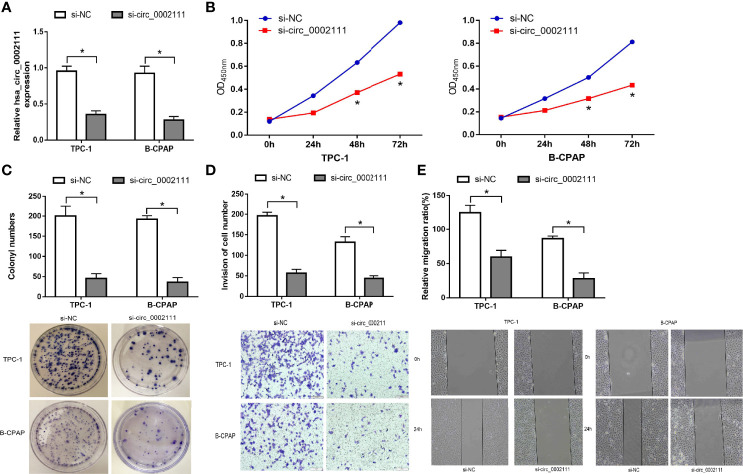
Inhibition of hsa_circ_0002111 suppresses the proliferation and invasion abilities of papillary thyroid cancer (PTC) cells. **(A)** The effect of si-circ_0002111 on TPC-1 and B-CPAP cells was determined by quantitative real-time polymerase chain reaction (qRT-PCR). **(B)** Cell counting kit-8 (CCK-8) assay was used to explore the cell proliferation ability of TPC-1 and B-CPAP cells transfected with si-circ_0002111. **(C)** Colony formation assay was performed to determine the colony-forming ability of TPC-1 and B-CPAP cells after transfection with si-circ_0002111. **(D, E)** Transwell and wound healing assays showed that TPC-1 and B-CPAP cells transfected with si-circ_0002111 led to reduced migration and invasion abilities. *P < 0.05.

To explore the role of hsa_circ_0002111 in PTC, TPC-1 and B-CPAP cells were transfected with si-circ_0002111 or si-NC. qRT-PCR revealed that hsa_circ_0002111 expression was significantly down-regulated in PTC cells after transfection of si-circ_0002111 (**Figure 4A**; P < 0.05). CCK-8 and colony formation assays both showed that the cell proliferation ability of TPC-1 and B-CPAP cells transfected with si-circ_0002111 was significantly reduced compared with that in the si-NC group (**Figures 4B, C**; P < 0.05). Furthermore, we used wound healing and transwell assays to explore the effect of hsa_circ_0002111 on the migration and invasion abilities of the PTC cells. Our data indicated that the inhibition of hsa_circ_0002111 significantly reduced the migration and invasion abilities of both TPC-1 and B-CPAP cells (**Figures 4D, E**; P < 0.05). These results suggested that hsa_circ_0002111 could function as an oncogenic circRNA in the progression of PTC.

## Discussion

circRNAs exist in various organisms and cells, including human cells (7). With rapid advances in next-generation sequencing technologies, many circRNAs have been identified in human transcriptomes (10–12), and subsequently, many circRNAs have been associated with a variety of cancers. Recent reports have revealed the critical roles of circRNAs in various biological processes, such as mediation of gene expression by sponging microRNAs and RBPs (RNA binding proteins), as well as regulation of transcription and alternative splicing (13, 14). Moreover, as circRNAs are more stable than other types of RNAs (9), they may serve as potential biomarkers and therapeutic targets for cancers. For instance, Zhong et al (15). showed that hsa_circ_0000993 could inhibit metastasis of gastric cancer by sequestering microRNA miR-214-5p. Wang et al (16). predicted that circRNA-000911 might play an anti-oncogenic role in breast cancer, and might serve as a promising therapeutic target for patients with breast cancer. With respect to PTC, the studies on circRNAs are in their infancy, and only a few circRNAs have been investigated, such as hsa_circ_0058124 (17) and circBACH2 (18). Besides, Peng et al (19). showed that hsa_circ_0002111 was one of sixteen significantly circRNAs upregulated in PTC tumors as compared to benign thyroid tissue with microarray profiling, Guo et al (20). demonstrated this result and found hsa_circ_0002111 was significantly associated with the BRAF^V600E^ mutation. But, the detailed role of hsa_circ_0002111 as a biomarker for the diagnostic and evaluation of progression of PTC wasn’t explored and validated in their studies. Thus, it is essential to elucidate the association between hsa_circ_0002111 expression and PTC development.

In the current study, we reported that hsa_circ_0002111 contributed to PTC progression, and could serve as a therapeutic target for PTC. We used qRT-PCR to analyse hsa_circ_0002111 expression in 82 pairs of PTC and adjacent normal tissues, and in normal human thyroid and PTC cell lines. The results indicated that hsa_circ_0002111 expression was significantly up-regulated in PTC tissues and cell lines. Notably, we found that high hsa_circ_0002111 expression was associated with an advanced TNM stage and lymph-node metastasis in PTC patients. Additionally, the AUC showed that hsa_circ_0002111 had the potential to be a biomarker for the diagnosis of PTC. We also explored the relationship between hsa_circ_0002111 and PTC progression using CCK-8, colony formation, transwell, and wound healing assays. Our results revealed that inhibition of hsa_circ_0002111 significantly suppressed the proliferation, migration, and invasion of PTC cells.

The limitations of our study must be acknowledged. First, we had a relatively small sample size and only detected the expression of hsa_circ_0002111 in tissue samples, our results would be more insightful if it is validated in a larger cohort together with the detection in the circulation of patients with PTC. Second, our research was mainly done in cell-based assays, so the function of hsa_circ_0002111 remains to be verified *in vivo*. Finally, the regulatory mechanism of hsa_circ_0002111 was not explored in this study. The abovementioned limitations warrant further investigations into the role of hsa_circ_0002111 in PTC.

In conclusion, the results of this study showed that hsa_circ_0002111 expression was markedly up-regulated in PTC tissues and cells, and closely associated with an advanced TNM stage and lymph-node metastasis. Additionally, inhibition of hsa_circ_0002111 expression suppressed the proliferation, migration, and invasion of PTC cells *in vitro*. Thus, these findings indicate that hsa_circ_0002111 may be a potential diagnostic and therapeutic target for PTC.

## Data Availability Statement

Publicly available datasets were analyzed in this study. This data can be found here: NCBI GEO Datasets (http://www.ncbi.nlm.nih.gov/gds, No. GSE93522).

## Ethics Statement

The studies involving human participants were reviewed and approved by The Ethics Committee of Scientific Research and Clinical Trial, The First Affiliated Hospital of Zhengzhou University. The patients/participants provided their written informed consent to participate in this study.

## Author Contributions

DY conceived of the idea and provided guidance. GD wrote the manuscript and completed the figures. KF, DN, RM, and JH carefully reviewed the manuscript. HL made critical revisions to the manuscript. All authors contributed to the article and approved the submitted version.

## Funding

This study was funded by The University Scientific and Technological Innovation Team Project of Henan Province (19IRTSTHN002), The Thousand Talents Science and Technology Innovation Leading Talents Subsidy Project of Central Plains (194200510011), Major Scientific Research Projects of Traditional Chinese Medicine in Henan Province (No.20-21ZYZD14), and Cultivation of Young and Middle-aged Health Science and Technology Innovation Leading Talents in Henan Province (YXKC2020015).

## Conflict of Interest

The authors declare that the research was conducted in the absence of any commercial or financial relationships that could be construed as a potential conflict of interest.
